# Comparative analysis of the *Geobacillus* hemicellulose utilization locus reveals a highly variable target for improved hemicellulolysis

**DOI:** 10.1186/1471-2164-15-836

**Published:** 2014-10-01

**Authors:** Pieter De Maayer, Phillip J Brumm, David A Mead, Don A Cowan

**Affiliations:** Centre for Microbial Ecology and Genomics, Genomics Research Institute, Natural Sciences II Building, University of Pretoria, Pretoria, 0002 South Africa; Department of Microbiology and Plant Pathology, University of Pretoria, Pretoria, 0002 South Africa; C5-6 Technologies Inc., Lucigen Corp, 53562 Middleton, WI USA

**Keywords:** *Geobacillus*, Hemicellulose, Arabinoxylan, Arabinan, Xylanase, Acetylesterase, Arabinofuranose

## Abstract

**Background:**

Members of the thermophilic genus *Geobacillus* can grow at high temperatures and produce a battery of thermostable hemicellulose hydrolytic enzymes, making them ideal candidates for the bioconversion of biomass to value-added products. To date the molecular determinants for hemicellulose degradation and utilization have only been identified and partially characterized in one strain, namely *Geobacillus stearothermophilus* T-6, where they are clustered in a single genetic locus.

**Results:**

Using the *G. stearothermophilus* T-6 hemicellulose utilization locus as genetic marker, orthologous hemicellulose utilization (HUS) loci were identified in the complete and partial genomes of 17/24 *Geobacillus* strains. These HUS loci are localized on a common genomic island. Comparative analyses of these loci revealed extensive variability among the *Geobacillus* hemicellulose utilization systems, with only seven out of 41–68 proteins encoded on these loci conserved among the HUS^+^ strains. This translates into extensive differences in the hydrolytic enzymes, transport systems and metabolic pathways employed by *Geobacillus* spp. to degrade and utilize hemicellulose polymers.

**Conclusions:**

The genetic variability among the *Geobacillus* HUS loci implies that they have variable capacities to degrade hemicellulose polymers, or that they may degrade distinct polymers, as are found in different plant species and tissues. The data from this study can serve as a basis for the genetic engineering of a *Geobacillus* strain(s) with an improved capacity to degrade and utilize hemicellulose.

**Electronic supplementary material:**

The online version of this article (doi:10.1186/1471-2164-15-836) contains supplementary material, which is available to authorized users.

## Background

The plant cell wall consists of four major structural polymer components, namely cellulose, hemicellulose, pectin and lignin [1, 2]. In contrast to cellulose, which is a homopolymer of β-(1 → 4)-linked glucose monomers, hemicelluloses incorporate a wide range of heterogeneous polymers containing a backbone of xylose, arabinose, galactose or, mannose monomers [1, 3]. The most common hemicelluloses are the xylans, which have a backbone of β-(1 → 4)-linked xylose monomers, and are frequently decorated with side chains of arabinose, glucuronic acid or its 4-O-methyl derivative, and acetyl side groups [1]. The structure of the hemicellulose, its backbone monomers and degree of branching and type of side chains varies extensively between different plant species and even tissues within the same plant. For example, xyloglucans dominate the primary cell walls of dicotyledonous plants, while glucuronoarabinoxylans are prevalent in both the primary and secondary cell walls of commelinid monocots (e.g. sugarcane and maize). While the primary cell walls of conifers such as pine are dominated by xyloglucans, the hemicellulose fraction of their secondary cell walls is comprised mainly of galactoglucomannans [1]. Similarly, pectin represents a family of heterogeneous polymers exhibiting complex structures which are found in the plant primary cell wall [4].

In nature, plant biomass represents the most abundant carbohydrate source for microorganisms. As such, many bacteria, fungi and yeast can degrade hemicellulose polymers through the production of a battery of hydrolytic enzymes, including xylanases, xylosidases, arabinofuranosidases and glucuronidases [5]. In recent decades there has been increasing interest in the use of microorganisms and their enzymes for the hydrolysis of hemicelluloses, as a result of the various biotechnological and industrial applications in which the hydrolytic enzymes, the hemicelluloytic process and its products can be exploited. For example, xylanases can be applied in the bleaching of pulp and paper, increasing the digestibility of animal feeds, clarification of fruit juices and the bioconversion of plant bionass [5–7]. The latter can be exploited, in combination with the fermentative capacities of microorganisms in the conversion of lignocellulosic biomass to high yields of the renewable energy source bioethanol and other value-added products [8, 9]. The oligosaccharides generated from hemicellulose hydrolysis can also be applied as prebiotics that inhibit harmful microorganisms and stimulate the growth of bifidobacteria and lactobacilli in the human intestine [10]. There has been increasing interest in the use of thermophilic microorganisms for the hemicellulytic hydrolysis of plant biomass, as they display a number of advantages over mesophilic microorganisms [8, 11]. The rate of biomass conversion is enhanced at higher growth temperatures. Furthermore, as high temperatures are required for the pre-treatment and delignification of plant biomass in many industrial processes, the ability of thermophilic microorganisms to tolerate higher temperatures, as well as producing thermostable hydrolytic enzymes, can potentially reduce process costs [8, 11].

Obligately thermophilic species within the genus *Bacillus* have been accommodated in the genus *Geobacillus*, which currently encompasses nineteen species [12, 13]. Members of this genus are rod-shaped, aerobic endo-spore formers and have optimum growth temperatures of between 45 and 75°C [13]. *Geobacillus* spp. serve as an attractive resource for production of a wide range of thermostable enzymes for a variety of biotechnological applications [8, 14]. *Geobacillus* strains have been shown to be highly effective in the degradation of hemicellulose polymers, and considerable attention has focused on the thermostable extra- and intra-cellular xylanases and β-xylosidases [15–18]. The molecular determinants underlying the capacity of this genus to utilize hemicellulose have, however, been largely elucidated in only one strain, *Geobacillus stearothermophilus* T-6, and have been shown to reside on a single genetic locus [19–21]. On the basis of characterization of some of the gene clusters within this locus, a model for the hemicellulolytic capacity of this strain has been developed [19–21]. The model hemicellulose substrate, acetylglucuronoarabinoxylan (GAX), is cleaved by the extracellular GH10 xylanase XynA1 into large xylooligomers, including xylooligosaccharides and aldotetraouronic acid, which are subsequently transport across the cellular membrane by specific oligosaccharide transport systems [19, 20]. The glucuronic acid is cleaved from the aldotetrauronic acid (2-*O*-α-(4-*O*-methyl-D-glucuronosyl)-xylotriose or MeGlcAXyl3) polymer by the action of a GH67 α-glucuronidase and is metabolized via the uronate metabolic pathway, also encoded in the hemicellulose utilization locus [19]. The xylooligosaccharides and xylotriose derived from MeGlcAXyl3 polymers are further cleaved into D-xylose monomers through the concerted action of a second intracellular GH10 xylanase, XynA2, and three β-xylosidases XynB1, XynB2 and XynB3, which are then metabolized via the pentose phosphate and glycolysis pathways [19, 20]. Pathways for the hydrolysis and utilization of arabinofuranosyl substituents and for the main chain deacetylation have also been described [20, 22, 23]. A gene cluster within the hemicellulose utilization locus of *G. stearothermophilus* T-6 has also been shown to play a role in the degradation of the pectin-associated L-arabinan polymer [21], further highlighting the capacity of *Geobacillus* spp. to degrade and utilize polymers in plant biomass.

To date, the genomes of twenty-four *Geobacillus* spp. have been sequenced. Here, using the *G. stearothermophilus* T-6 hemicellulose utilization system (HUS) as a model, we have identified and characterized loci coding for the degradation and utilization of hemicellulose in most of the sequenced strains. Our analyses reveal the extensive variability in the *Geobacillus* hemicellulose utilization loci, which could be exploited to generate recombinant strains with improved hemicellulolytic capacities.

## Results and discussion

### A hemicellulose utilization system is a common feature among most *Geobacillus*spp

**Figure 1:**
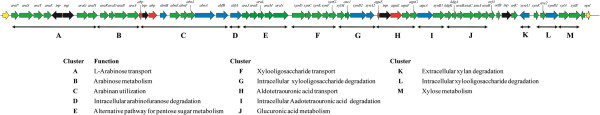
**Schematic diagram of the** ***G. stearothermophilus*** **T**-**6 hemicellulose utilization locus.** Each arrow represents a gene in the locus, with genes encoding predicted transposons colored in black, while open reading frames interrupted by transposons are colored in red. Genes encoding glycosyl hydrolases are colored in blue. The *G. stearothermophilus* HUS locus was subdivided into thirteen gene clusters on the basis of their predicted function.

The capacity of *G. stearothermophilus* T-6 to degrade and utilize hemicellulose is dependent on a single complex gene locus, which has recently been sequenced (NCBI Acc. # DQ868502). This locus is ~76 kb in size, has a G + C content of 46.93% and encodes 60 proteins. The HUS locus of T-6 can be roughly divided into thirteen distinct gene clusters (Figure 1 – clusters A-M). Twelve of the gene clusters have been characterized functionally and/or *in silico* and have been shown to play roles in the concerted degradation and utilization of the hemicellulose polymer glucuronoarabinoxylan in this strain [19, 20]. Of these, seven can be considered as central to hemicellulose degradation, with the proteins encoded in these clusters driving the extracellular degradation of arabinoglucuronoxylan (cluster K), transport of the cleaved oligosaccharide products into the cell (clusters F and H) and further intracellular degradation of these intermediates into metabolizable monosaccharides (clusters D, G, I and L). Four additional gene clusters (clusters B, E, J and M) are not essential for the degradation of the hemicellulose polymer, but rather encode pathways for the metabolism of the end-product pentose sugars arabinose and xylose and for uronic acids, as well as a putative alternative pathway for pentose sugar metabolism [19, 20]. An additional cluster which is not essential for hemicellulose degradation, encodes a transporter of L-arabinose monosaccharides (cluster A). A gene cluster with a role in the degradation and utilization of the pectin-associated polymer L-arabinan has also been described in *G. stearothermophilus* T-6 [21] and is positioned in the hemicellulose utilization cluster of this strain (Figure 1 - cluster C).

**Figure 2:**
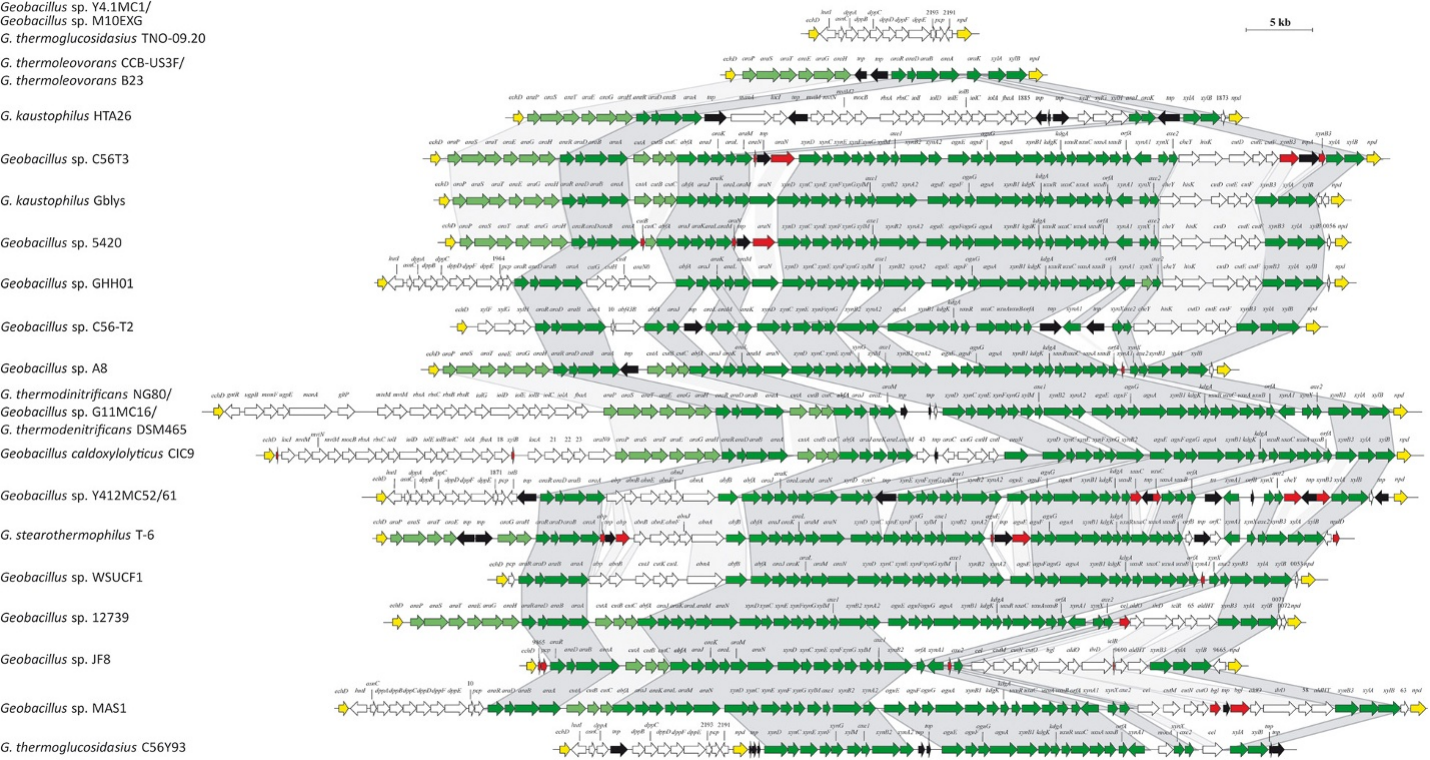
**Comparative diagram of the** ***Geobacillus*** **hemicellulose loci.** The aligned *echD*-*npd* islands of the twenty-four sequenced HUS^+^ and HUS^-^ *Geobacillus* strains and that of *G. stearothermophilus* T-6 are shown. The flanking *echD* and *npd* genes are colored in yellow, genes coding for transposons in black, and genes in which the reading frames are transposon-disrupted are in red. Those genes conserved among >70% of the HUS^+^ strains are colored in green, those conserved among >50% and <70% in light green, while those conserved among <50% of the HUS^+^ strains are colored in white.

The genomes of 24 *Geobacillus* strains, thirteen complete and eleven draft genomes, have been sequenced (Table 1) [24–34]. The twelve gene clusters of T-6 with a reported role in hemicellulose utilization were used as units for the identification of orthologous clusters in the sequenced *Geobacillus* genomes, by localized BlastN analysis. Between three and twelve partial or complete orthologous clusters were found in twenty of the sequenced strains, while no orthologous clusters could be found in the genomes of the remaining four strains (Table 2; Figure 2). A further three strains, namely *G. thermoglucosidasius* CCB_US3_UF5 and B23, as well as *G. kaustophilus* HTA426 only carry three orthologous gene clusters, namely those for xylose metabolism, arabinose transport and L-arabinose metabolism. The lack of orthologs of all T-6 clusters required for hemicellulose degradation suggests that these strains do not have this capacity, and rather make use of L-arabinose and D-xylose monomers that may be present in the environment. The remaining seventeen sequenced *Geobacillus* strains contain between eight and twelve gene clusters sharing orthology with the thirteen clusters that make up the hemicellulose utilization locus of *G. stearothermophilus* T-6 (Table 2), including between five and seven of the clusters with a role in hemicellulose degradation. The genomic locations of the orthologous clusters indicate that, as is the case of T-6, they are arranged into a single chromosomal hemicellulose utilization locus. Thus, including *G. stearothermophilus* T-6, eighteen *Geobacillus* strains (72% of the twenty-five strains analyzed) can be considered to contain a Hemicellulose Utilization System (HUS). In addition to T-6, the genomes of three further sequenced *Geobacillus* strains, namely Y412MC52, Y412MC61 and WSUCF1, were observed to encode an L-Arabinan Utilization System (AUS) incorporated within the hemicellulose utilization locus.

**Table 1:** **Genome characteristics of twenty-four sequenced** ***Geobacillus*** **strains**

Species	Strain	Isolation source	Sequencing status	NCBI Acc #	Replicons/contigs	Size (Mb)	G + C%	Ref
*G. thermoleovorans*	B23	Subterraneon oil reservoir, Japan	Draft	BATY00000000	209	3.35	52.29	[24]
*G. thermoleovorans*	CCB_US3_UF5	Ulu Slim hot spring, Malaysia	Complete	NC_016593.1	1	3.60	52.28	[25]
*G. kaustophilus*	HTA426	Deep sea sediment, Marian Trench	Complete	BA000043.1	2	3.59	51.98	[26]
*Geobacillus* sp.	CAMR5420	-	Draft	JHUS01000000	96	3.49	52.00	[27]
*G. kaustophilus*	GBlys	Pasteurized milk	Draft	BASG00000000	216	3.54	52.05	[28]
*Geobacillus* sp.	MAS1	Hot spring, Pakistan	Draft	AYSF00000000	121	3.50	52.21	-
*Geobacillus* sp.	A8	Deep mine water, South Africa	Draft	AUXP01000000	173	3.35	52.41	-
*Geobacillus* sp.	CAMR12739	-	Draft	JHUR01000000	74	3.38	52.19	[27]
*Geobacillus* sp.	C56-T3	Sandy’s Spring W, Nevada, USA	Complete	CP002050.1	1	3.65	52.49	-
*Geobacillus* sp.	Y412MC61	Obsidian Hot Spring, YNP, USA	Complete	NC_013411.1	2	3.67	52.33	-
*Geobacillus* sp.	Y412MC52	Obsidian Hot Spring, YNP, USA	Complete	NC_014915.1	2	3.67	52.33	-
*Geobacillus* sp.	WSUCF1	Compost, USA	Draft	ATCO00000000	346	3.40	52.21	[29]
*Geobacillus* sp.	GHH01	Soil, Germany	Complete	NC_020210.1	1	3.58	52.28	[30
*Geobacillus* sp.	C56-T2	Double Hot Springs, Nevada	Draft	SAMN0017395	3	3.55	52.39	-
*Geobacillus* sp.	JF8	Compost, Japan	Complete	NC_022080.4	2	3.49	52.79	[31]
*Geobacillus* sp.	G11MC16	-	Draft	ABVH00000000	31	3.55	48.80	-
*G. thermodenitrificans*	NG80-2	Subterraneon oil reservoir, China	Complete	NC_009328.1	2	3.61	48.86	[32]
*G. thermodenitrificans*	DSM465^T^	Sugar beet juice, Austrua	Partial	AYKT00000000	76	3.40	49.05	[33]
*G. caldoxylolyticus*	CIC9	Hot Spring, Indonesia	Partial	AMRO0000000	82	3.83	44.17	-
*G. thermoglucosidasius*	C56YS93	Obsidian Hot Spring, YNP, USA	Complete	NC_015660.1	3	4.00	43.93	-
*G. thermoglucosidasius*	TNO-09.020	Dairy processing plant, Netherlands	Complete	NZ_CM001483	1	3.74	43.82	[34]
*G. thermoglucosidasius*	M10EXG	Waste-composting unit, Australia	Complete	2501416905^a^	1	3.67	43.74	-
*Geobacillus* sp.	Y4.1MC1	Bath Hot Spring, YNP, USA	Complete	NC_014650.1	2	3.84	44.02	-
*Geobacillus* sp.	WCH70	Middleton, USA	Complete	NC_012793.1	3	3.51	42.80	-

NCBI Acc # refers to the NCBI accession numbers of the genomes for the specified organisms. ^a^refers to the Integrated Microbial Genomes (IMG) object identifier for the genome of *G. thermoglucosidasius* M10EXG ( http://img.jgi.doe.gov).

**Table 2:** **The number (#) of orthologous gene clusters from the** ***G*** **.** ***stearothermophilus*** **T-6 HUS loci in sequenced** ***Geobacillus*** **strains**

Strain number	*Geobacillus*strain	# orthologous clusters
1	*G. thermoleovorans* B23	3
2	*G. thermoleovorans* CCB_US3_UF5	3
3	*G. kaustophilus* HTA426	3
4	*Geobacillus* sp. CAMR5420	12
5	*G. kaustophilus* GBlys	12
6	*G. thermopakistaniensis* MAS1	11
7	*Geobacillus* sp. A8	12
8	*Geobacillus* sp. CAMR12739	12
9	*Geobacillus* sp. C56-T3	12
10	*Geobacillus* sp. Y412MC61	12
11	*Geobacillus* sp. Y412MC52	12
12	*Geobacillus* sp. WSUCF1	12
13	*Geobacillus* sp. GHH01	11
14	*Geobacillus* sp. C56-T2	10
15	*Geobacillus* sp. T-6	12
16	*Geobacillus* sp. JF8	8
17	*Geobacillus* sp. G11MC16	12
18	*G. thermodenitrificans* NG80-2	12
19	*G. thermodenitrificans* DSM465^T^	12
20	*G. caldoxylolyticus* CIC9	11
21	*G. thermoglucosidasius* C56YS93	8
22	*G. thermoglucosidasius* TNO-09.020	0
23	*G. thermoglucosidasius* M10EXG	0
24	*Geobacillus* sp. Y4.1MC1	0
25	*Geobacillus* sp. WCH70	0

**Figure 3:**
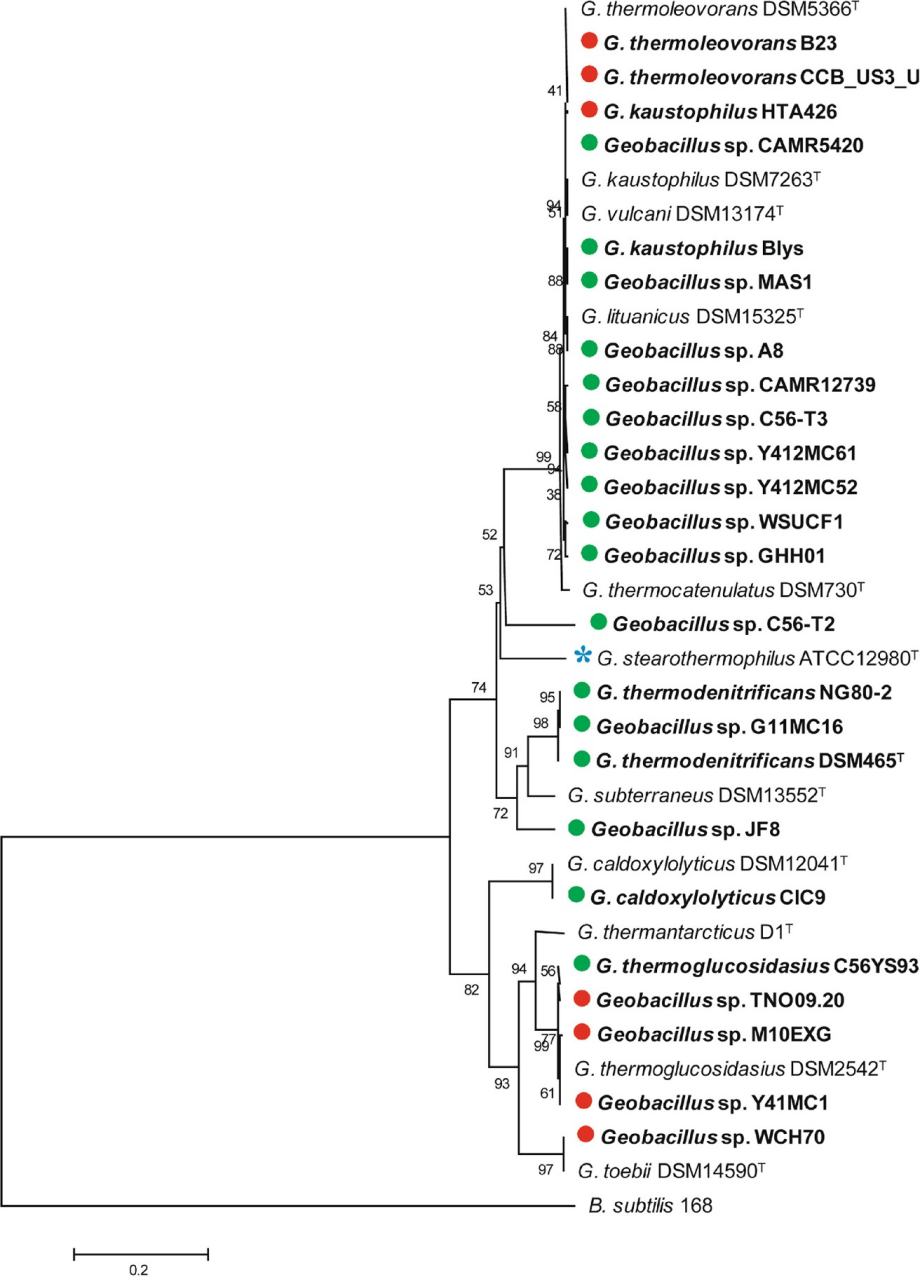
**Phylogeny of the sequenced** ***Geobacillus*** **strains showing the distribution of hemicellulose loci.** A neighbour-joining phylogeny was constructed on the basis of the *recN* gene for the twenty-four sequenced *Geobacillus* strains as well as twelve *Geobacillus* spp. type strains. The presence or absence of HUS loci in the sequenced strains is indicated by green and red dots, respectively. The blue star next to *G. stearothermophilus* ATCC 12980^T^ indicates the predicted phylogenetic position of *G. stearothermophilus* T-6 for which a *recN* sequence is not available. The *recN* gene sequence of *Bacillus subtilis* 168 was used as outgroup. Bootstrap values (n = 1,000) are shown.

A phylogenetic tree including all sequenced *Geobacillus* spp. and twelve *Geobacillus* spp. type strains was constructed using the *recN* gene. Phylogeny using the *recN* gene has been demonstrated to have great resolving power at the species and subs-species level for the genus *Geobacillus* [35]. The topology (Figure 3) showed that the hemicellulose utilization system is encoded across all major branches of the *Geobacillus* genus phylogeny, although clusters of HUS^-^ strains can be observed in the *G. thermoleovorans*-*kaustophilus* and *G. thermoglucosidasius*-*thermantarcticus*-*toebii* branches.

### The *Geobacillus*hemicellulose system forms part of a genomic island

Genomic islands were identified in the genomes of the thirteen complete *Geobacillus* genomes using the Islandviewer server [36]. Between thirteen and thirty genomic islands were predicted for each strain. One predicted island, flanked by genes encoding an enoyl-CoA hydratase (*echD*) and a nitropropane dioxygenases (*npd*), is present on the chromosome of twelve out of thirteen complete sequenced *Geobacillus* strains. BlastN analysis with these genes identified similar islands in all eleven draft genomes (Figure 2), suggesting that an *echD*-*npd* island is present in twenty-three of the twenty-four sequenced strains. While orthologs of the *echD* and *npd* genes were detected in the genome sequence of *Geobacillus* sp. WCH70, these are separated by ~540 kb of sequence and were not identified as an island. The *echD*-*npd* islands range in size from 10.5 to 87.6 kilobases and carry between eleven and sixty-eight protein coding sequences (Table 3). The G + C content of the *echD*-*npd* island in all *Geobacillus* strains is on average 4.90 ± 1.15% (average G + C content ± standard deviation) lower than the genomic G + C content, indicative of horizontal acquisition of this island.

**Table 3:** **Characteristics of the** ***echD*** **-** ***npd*** **islands of twenty-five** ***Geobacillus*** **strains**

Strain #	Species	Strain	Island size (kb)	# CDS	G + C%	G + C% island vs genome
1	*G. thermoleovorans*	B23	22.0	17	48.24	-4.05
2	*G. thermoleovorans*	CCB_US3_UF5	22.0	16	48.27	-4.01
3	*G. kaustophilus*	HTA426	54.9	41	48.04	-3.94
4	*Geobacillus* sp.	CAMR5420	66.4	53	46.54	-5.46
5	*G. kaustophilus*	GBlys	68.1	53	46.20	-5.85
6	*Geobacillus* sp.	MAS1	74.7	62	46.12	-6.09
7	*Geobacillus* sp.	A8	60.4	48	46.75	-5.66
8	*Geobacillus* sp.	CAMR12739	67.9	54	46.65	-5.54
9	*Geobacillus* sp.	C56-T3	69.7	55	46.49	-6.00
10	*Geobacillus* sp.	Y412MC61	79.4	64	47.28	-5.05
11	*Geobacillus* sp.	Y412MC52	79.4	64	47.28	-5.05
12	*Geobacillus* sp.	WSUCF1	58.3	47	46.86	-5.35
13	*Geobacillus* sp.	GHH01	71.5	57	46.29	-5.99
14	*Geobacillus* sp.	C56-T2	60.8	44	47.00	-5.39
15	*Geobacillus stearothermophilus*	T-6	76.1	60	46.92	-
16	*Geobacillus* sp.	JF8	50.7	41	46.16	-6.63
17	*Geobacillus* sp.	G11MC16	86.3	68	43.73	-5.07
18	*G. thermodenitrificans*	NG80-2	86.4	68	43.75	-5.11
19	*G. thermodenitrificans*	DSM465^T^	86.4	68	43.76	-5.29
20	*G. caldoxylolyticus*	CIC9	87.6	68	38.83	-5.34
21	*G. thermoglucosidasius*	C56YS93	12.4	12	41.89	-2.04
22	*G. thermoglucosidasius*	TNO-09.020	10.6	11	40.69	-3.13
23	*G. thermoglucosidasius*	M10EXG	10.5	11	40.65	-3.09
24	*Geobacillus* sp.	Y4.1MC1	10.6	11	40.56	-3.46
25	*Geobacillus* sp.	WCH70	-	-	-	-

BlastN analysis with the *echD* and *npd* genes from the complete genomes against the *G. stearothermophilus* T-6 HUS locus revealed that a complete copy of *echD* and a partial *npd* gene were present at the 5′ and 3′ ends of this locus, respectively. A search up- and downstream of the hemicellulose degradation and utilization gene clusters in sixteen of the sequenced HUS^+^ strains showed that, similarly, all of the hemicellulose utilization loci in these strains were maintained within the *echD*-*npd* genomic islands, The three *Geobacillus* strains which contained orthologs of only three of the thirteen clusters in the T-6 hemicellulose utilization locus also carry these clusters within the *echD*-*npd* island, indicating that the *echD*-*npd* genomic islands serve as a centralized repository for the hemicellulose degradation and utilization genes within *Geobacillus* spp. One exception is the HUS^+^ strain *G. thermoglucosidasius* C56YS93, where the orthologous clusters for hemicellulose degradation and utilization are localized downstream of the *npd*-*echD* island and are flanked by transposon genes (Figure 2).

The average difference in G + C content between the *echD*-*npd* island and the genome is 3.61 ± 0.41% for HUS^-^ strains, while the mean difference for HUS^+^ is somewhat larger (5.55 ± 0.44%). An exception is again observed for *G. thermoglucosidasius* C56YS93, where the G + C content of the *echD*-*npd* island is only 2.04% below that of the genome, while that of the island plus the HUS locus is 0.06% above that the genome. Of note is the great variability in genomic G + C contents observed for the different sequenced *Geobacillus* strains. For example, the mean G + C content of the *G. caldoxylolyticus* CIC9 genome is 44.17%, and that of *Geobacillus* sp. A8 is 52.41%, while the mean G + C contents of their *echD*-*npd* islands are 46.75% and 38.83%, respectively. We suggest that these differences, given that the islands are highly variable, might be due to the non-conserved content of the island. The mean G + C contents for 41 conserved genes and those of the non-conserved genes in the *echD*-*npd* islands of these two strains were determined. This showed that G + C content differences existed between the islands of the A8 and CIC9 strains for both conserved and non-conserved genes, but that there was negligible variation in the G + C contents of both the conserved and non-conserved gene complement in the two strains (Additional file 1: Table S1). The concept of G + C amelioration [37] implies that while the G + C contents of genomic islands that have recently been horizontally acquired reflect those of the donor strain(s), the genes in these islands are subject to the same mutational processes as all other genes in the recipient strain and will become more similar to that of the recipient genomes over time. We suggest that the observed differences of genomic and island G + C contents among *Geobacillus* strains is due to the fact that the *echD*-*npd* islands may have derived from a common ancestor with a much lower genomic G + C content, and that these islands show G + C contents which are more, or less, ameliorated, depending on the time since acquisition.

### Comparison of the hemicellulose utilization loci reveals extensive variation among *Geobacillus*strains

**Figure 4:**
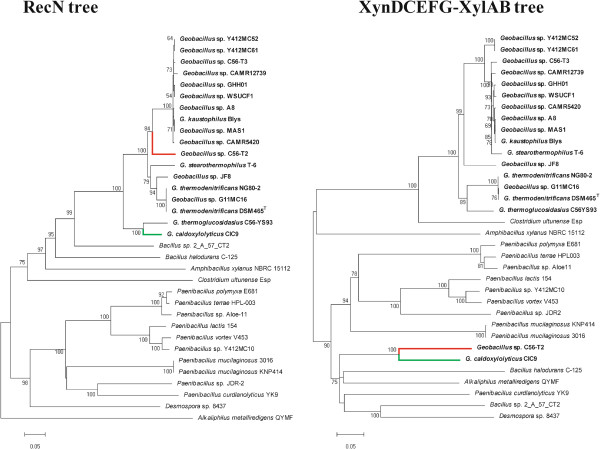
**Phylogenetic comparison of the HUS** ^**+**^ ***Geobacillus*** **strains on the basis of RecN and XynDCEFG-XylAB.** A neighbour-joining tree was constructed on the basis of the RecN amino acid sequences of the HUS^+^ *Geobacillus* strains as well as sixteen representatives of related genera in which orthologs of XynDCEFG-XylAB are present. Similarly a neighbour-joining tree was constructed based on alignment of the concatenated XynDCEFG-XylAB amino acid sequences. *Desmospora* sp. 8437 was used as outgroup and bootstrap values are shown (n = 1,000). The branches in red and in green, represent the phylogenetic positions of *Geobacillus* sp. C56-T2 and *G. caldoxylolyticus* CIC9, respectively, in both trees.

The protein coding sequences (CDSs) on the *echD*-*npd* islands of all twenty-five *Geobacillus* strains were predicted using FgenesB [38]. The translated CDS sets from both HUS^-^ and HUS^+^ strains were compared by localized BlastP analyses and the proteins were annotated on the basis of orthologous proteins identified by comparison against the NCBI non-redundant protein database (Additional file 2: Table S2). No proteins were conserved among the *echD*-*npd* islands of all twenty-five analyzed strains, while only seven proteins are conserved among the HUS^+^ strains. As between 41 and 68 proteins are encoded on the *echD*-*npd* islands of HUS^+^ strains, the low number of conserved proteins (10.3-17.1% of the total proteins encoded in the islands) highlights the extensive variability among the *Geobacillus* HUS loci. CDSs conserved among the eighteen HUS^+^ strains include two coding for a two component system (*xynDC*), three coding for a xylotriose transporter (*xynEFG*) and two genes (*xylAB*) required for xylose metabolism. The translated proteins products share an average amino acid identity of 87.35% between pair-wise compared strains, although lower orthology is observed for the XynDCEFG-XylAB sets of *G. caldoxylolyticus* CIC9 and *Geobacillus* sp. C56-T2, which share 60.5% and 67.4% average amino acid identity with each of the other sixteen HUS^+^ strains, respectively, and 80.7% amino acid identity between them. A neighbour-joining phylogeny was constructed on the basis of the concatenated XynDCEFG-XylAB amino acid sequences of the eighteen HUS^+^*Geobacillus* strains, as well as sixteen phylogenetic relatives for which whole genome sequences are available and in which orthologous proteins are present. The resultant tree (Figure 4) shows congruence in the clustering of most HUS^+^*Geobacillus* strains with the associated RecN tree, with the exception of *Geobacillus* sp. C56-T2 and *G. caldoxylolyticus* CIC9, suggesting that the hemicellulose utilization loci of sixteen strains have been derived from a common ancestor and have subsequently diverged in parallel with their genomes, while those of the other two strains are derived from a separate, but possibly common, ancestor.

#### Variability in the extracellular hydrolytic enzyme complement encoded on the Geobacillus HUS loci

The subcellular localizations of all the HUS-encoded CDSs were predicted by comparison of their translated protein products against the PsortB 3.0 server [39] and the detection of signal peptides by submission to the SignalP 4.1 server [40]. SignalP analysis predicted seventeen distinct HDI-encoded proteins with typical Gram-positive signal peptide/cleavage sites required for the Sec-dependent transport of proteins across the cellular membrane. PsortB analysis showed that the majority of these (fifteen out of seventeen proteins) are integrated into the cellular membrane and we propose that these form part of transport systems for the uptake of oligosaccharides. On the basis of Psort and SignalP results, two HUS-encoded proteins are predicted to be secreted. The protein sequences were compared against the Carbohydrate Active enzymes (CAZY) database using the dbCAN server’s BLAST tool [41, 42]. This showed that these two proteins represent glycoside hydrolases belonging to the Glycoside Hydrolase (GH) families GH10 and GH43, respectively.

Orthologs of a thermostable extracellular GH10 family xylanase XynA1 that degrades xylan to its component xylooligosaccharides have been identified in a number of *Geobacillus* spp. [15, 16, 43–45]. An orthologous *xynA1* gene is found in the hemicellulose utilization locus of 15/18 HUS^+^ strains. This gene is ~1,2 kb in size and is transcribed on the opposite strand to all other genes in the HUS loci. Truncated genes (223 nt) showing sequence homology to the 3′ end of the *xynA1* gene are also present in the HUS loci of *Geobacillus* sp. A8 and WSUCF1, while no orthologs is present in *G. caldoxylolyticus* CIC9 HDI, suggesting that the latter three strains lack the ability to degrade xylan extracellularly into its component xylooligosaccharides. The translated protein products of the complete *xynA1* genes share an average amino acid identity of 90.9% among the HDI^+^ and 90.5% to XynA1 of *G. stearothermophilus* T-6 (P40943; Bitscore: 765; e-value: 0.0). An extracellular GH43 endo α-1,5-arabinanase (*abnA*) has been shown to hydrolyze pectin-associated L-arabinan polymers into shorter arabino-saccharides and arabinose in *G. stearothermophilus* T-6 [21] and forms part of the AUS locus in this strain. Orthologs of *abnA* are also found in the other three AUS^+^ strains and their encoded proteins share 97.8% average amino acid identity to the T-6 arabinanase.

Additional predicted orthologous glycoside hydrolases are encoded in the HUS loci of *Geobacillus* sp. JF8, MAS1, and *G. thermoglucosidasius* C56 YS93, which share 93.9% average amino acid identity among them. The ~1.5 kb gene for this enzyme is localized in a non-conserved region of the HUS locus between the genes coding for a predicted acetyl-esterase (*axe2*) and a β-xylosidase (*xynB3*) (Figure 2). A truncated copy of this gene is also present within this region in *Geobacillus* sp. CAMR12739. Comparison of the translated protein products of the three complete gene copies against the CAZY database using the dbCAN Blast tool [41, 42] showed that they code for a GH5 glycoside hydrolase, showing greatest orthology to a glycoside hydrolase family protein in *Verminephrobacter eiseniae* EF01-2 (ABM56721.1; average amino acid identity: 57%; Bitscore: 593; e-value: 1e-168). The GH5 family includes enzymes active on a wide range of carbohydrate substrates, including chitosanases, β-mannosidases, xyloglucan-specific endo- β-1,4-glucanases and cellulases [46]. The target carbohydrate for a glycoside hydrolases can sometimes be further elucidated by the identification of carbohydrate binding modules (CBM), which promote the association of the enzyme with a particular substrate, within the protein sequence of the enzyme [47]. Comparison against the CAZY database using the dbCAN Blast tool identified a module showing weak orthology to a CBM family 6 module of a predicted endoglucanase in *Ignavibacterium album* JCM16511 (IALB_2578; Average amino acid identity: 31.4%; Bitscore: 157; e-value: 2e-35) at the N-terminal end (amino acid positions 32–285) of the GH5 protein orthologs of all four *Geobacillus* strains. The CBM6 modules have been demonstrated to have binding specificities towards a number of substrates, including both branched and debranched xylan, β-1,4-glucan (cellulose), and agarose [48]. It is thus difficult to distinguish the target carbohydrate substrate for the *Geobacillus* GH5 enzyme. Similarly, it is difficult to determine the subcellular localizations of these enzymes. SignalP analysis shows that no typical Gram-positive signal peptides are present. The PsortB SubCellular Localization SCL-BLAST + tool, however, showed that the *Geobacillus* proteins share orthology with the cellodextrinase CelA of *Ruminococcus flavefaciens* FD-1 (P16169), for which an extracellular localization has been determined experimentally [49].

The limited number of secreted hydrolytic enzymes has been proposed to provide a selective advantage to *Geobacillus* spp. and related hemicellulolytic organisms, as the relatively large oligosaccharides which result from extracellular hemicellulose hydrolysis will require further cleavage by intracellular enzymes and are thus not readily available to competing, non-hemicellulolytic, microorganisms [21]. The assimilation of xylo- and arabinoxylo-oligosaccharides may furthermore serve in the conservation of energy.

#### Variability in the membrane transport systems encoded on the Geobacillus HUS locus

Following the extracellular degradation of xylan into its component oligosaccharides, these intermediates are typically transported into the cell by means of specific ABC sugar transporters [21]. Carbohydrate Uptake Transporters (CUT) have been classified into two distinct families, with CUT1 signifying those transporters that facilitate the uptake of di- and oligosaccharides as well as glycerol phosphate and polyols, while members of the CUT2 family transport only monosaccharides [50, 51]. Eight gene clusters encoding distinct CUT1 transporters could be identified among the HUS^+^ strains, with between two and four of these present in each of the strains, while a single CUT2 family transporter is encoded in most of the HUS^+^ as well as three HUS^-^ strains.

Three CUT1 transporters with a role in transport of hemicellulose/pectic polymer degradation products have been described and functionally characterized in *G. stearothermophilus* T-6. These are required for the uptake of arabinosaccharides (*abnEFJ*), xylooligosaccharides (*xynEFG*) and aldotetraouronic acid (2-*O*-α-(4-*O*-methyl-D-glucuronosyl)-xylotriose – MeGlcAXyl3) (*aguEFG*) [19–21]. Orthologs of the *xynEFG* xylooligosaccharide transporter, which preferentially transports xylotriose, are found in all eighteen HUS^+^ strains, while the *aguEFG* aldotetraouronic acid transporter is present in sixteen HUS^+^ strains. The reading frame of the *aguE* gene coding for an aldotetraouronic acid extracellular sugar-binding protein in T-6 is disrupted by a transposon, suggesting that this transporter is non-functional in this strain. In contrast to the prevalent *aguEFG* and *xynEFG* transport systems, the *abnEFJ* arabinosaccharide transporter, which forms part of the L-arabinan utilization system, is restricted in distribution to three strains, namely T-6, Y412MC52 and Y412MC52. One CUT2 transporter has also been identified in the HUS locus of *G. stearothermophilus* T-6, which is predicted to transport arabinose monosaccharide [21]. The polycistronic operon (*araEGH*) coding for this transporter is also disrupted in T-6 by a transposon insertion. Orthologs are present in ten out of the 18 HDI^+^ strains, and also in three HDI^-^ strains, suggesting that this transporter is not a characteristic of hemicelluloses degrading strains, but may form a part of an arabinose metabolic pathway present in most *Geobacillus* spp.

The five remaining distinct CUT1 transporters encoded in the HUS loci of the different sequenced *Geobacillus* strains share only limited orthology to characterized transporters. For further reference, the gene clusters encoding these transporters were named *cutABC*, *cutDEF*, *cutGHI*, *cutJKL*, *cutMNO* in order to distinguish between them and to avoid duplicity of gene names (Figure 2; Additional file 1: Table S1). A locus in *Bacillus subtilis* encodes a CUT1 transporter for the uptake of α-1,5-arabinooligosaccharides [52, 53]. Orthologous loci (*cutABC*) are present in 10/18 HUS^+^ strains, all of which are missing *abnEFJ* orthologs, with the translated protein products sharing 58.4% average amino acid identity with the AraNPQ proteins of *B. subtilis* 168. In *Geobacillus* sp. CAMR5420, only a partial *cutB* and complete *cutC* gene are present, while an ortholog of *cutA* is missing. The *B. subtilis* AraNPQ transport system is required for the uptake of linear α-1,5-arabinosaccharide homopolymers consisting of three or four arabinosyl units residues, but not the branched arabinooligosaccharides decorated with α-1,2- and α-1,3-linked arabinosyl side chains. These branched arabinooligosaccharides are transported into the cell by an as yet unknown mechanism [53]. The nature of the substrate(s) transported by the *Geobacillus* CutABC system is yet to be experimentally determined. A distinct CUT1 transporter (*cutJKL*) is encoded within the AUS locus of *Geobacillus* sp. WSUCF1 in the location occupied by *abnEFG* in the other AUS^+^ strains. The translated products for these genes share only 43.7% average amino acid identity to *G. stearothermophilus* T-6 AbnEFJ, but share greater orthology (71.7% average amino acid identity) with an ABC transporter of an unknown sugar substrate in *Bacillus halodurans* C-125 (BH1864-1866). The location of the CUT-1 encoding genes between the arabinanase-encoding *abnA* and *abnB* genes in WSUCF1, however, suggests that as in the case of T-6, they encode an arabinosaccharide transporter, although whether this CUT1 system facilitates the uptake of linear or branched saccharides is unknown.

The *cutDEF* genes encoding a CUT1 transporter are located between the *axe2* and *xynB3* genes in the loci of 5/18 HUS^+^ strains. Their translated products share 63.7% average amino acid identity with a characterized aldotetrouronate transporter in *Paenibacillus* JDR-2 [54], but share only 33.4% average amino acid identity with the *aguEFG* aldotetrouronate transporter of *G. stearothermophilus* T-6. Four of the strains with a *cutABC* cluster also encode *aguEFG* orthologs, suggesting they possess two alternative pathways for the uptake of aldotetraouronic acid. On the other hand, C56-T2 lacks *aguEFG* orthologs, but does appear to carry a functional *cutDEF* system. The translated protein products of the predicted *cutMNO* CUT1 transporter (Figure 2) in the HUS loci of in *Geobacillus* sp. MAS1 and JF8 share 96.2% amino acid identity between the two strains but show limited orthology (36.4% average amino acid identity) to the *xynEFG* transporter in *G. stearothermophilus* T-6. The localization of the *cutMNO* genes adjacent to the gene encoding the predicted GH5 hydrolytic enzyme in these two strains, suggests they likely code for a transporter for the uptake of the degraded saccharide products of the latter enzyme. The other two strains which encode a GH5 ortholog, namely *Geobacillus* sp. CAMR12739 and *G. thermoglucosidasius* C56YS93 do not contain an orthologous transporter. Three genes encoding an additional orthologous CUT1 transporter (*cutGHI*) are localized between *araRDBA* and *abfA*-*araJKLMN* in *Geobacillus* sp. HH01 and between *araM* and *araN* in *G. caldoxylolyticus* CIC9, respectively (Figure 2). The encoded proteins share 85.8% average amino acid identity between these two strains and 72.5% average amino acid identity with a predicted transporter of an unknown sugar substrate in *Paenibacillus mucilaginosis* 3013 (PMI3016_2562-2564).

#### Variability in the intracellular enzyme complement of HUS^+^ Geobacillus spp

Typically, following the intracellular transport of the component oligosaccharides they are further degraded into monomeric sugars through the concerted action of a battery of intracellular glycosyl hydrolases [19, 20]. The intracellular enzyme complement for the cleavage of xylooligosacharides, methylglucuronate side chains and L-arabinan arabinosaccharides have been characterized in *G. stearothermophilus* T-6 [19–21]. The AguEFG-transported substrate MeGlcAXyl3 is cleaved into D-glucuronic acid and xylotriose by the GH67 family α-glucuronidase AguA [19, 55]. Orthologs of the T-6 *aguA* gene are located in the loci of all HUS^+^ strains, with the exception of *Geobacillus* sp. JF8. As this strain is also missing orthologs of *aguEFG* it is likely that this strain cannot utilize this substrate. The *Geobacillus* sp. C56-T2 HUS locus does encode an AguA ortholog, but is also missing orthologs of the *aguEFG* genes, suggesting this strain can also not utilize aldotetraouronic acid as a metabolic intermediate, although it cannot be excluded that an alternative transporter for this substrate may exist in this strain.

The xylotriose derived from cleavage of MeGlcUAX_3_, as well as the xylooligosaccharides transported into the cell via the *xynEFG* transporter, are degraded to D-xylose through the concerted action of an intracellular xylanase and β-xylosidases [19, 56]. The *xynA2* gene in *G. stearothermophilus* T-6 encodes an intracellular GH10 family xylanase that degrades xylooligosaccharides, including xylotriose, into xylose and xylobiose subunits [18]. Orthologs are encoded in all HUS^+^ strains with the exception of *Geobacillus* sp. JF8 and *G. caldoxylolyticus* CIC9. The structure, biochemistry and mechanism of action of three distinct β-xylosidases encoded in the HUS locus of *G. stearothermophilus* T-6, XynB1 (GH39), XynB2 (GH52) and XynB3 (GH43), have been described [17, 56–59]. The natural substrate of XynB2 and XynB3 is xylobiose, while that of XynB1 is xylotriose. The *xynB2* gene forms part of the xylooligosaccharide degradation cluster (Figure 1 - cluster G) of the loci of all eighteen HUS^+^*Geobacillus* strains, and as such probably degrades xylobiose released through the intracellular hydrolysis of *xynEFG*-transported xylooligosaccharides by *xynA2*. The *xynB1* gene forms part of the aldotetraouronic acid utilization cluster (Figure 1 – cluster I) and the ability of XynB1 to degrade xylotriose, the cleavage product of MeGlcUAX_3_, may preclude the requirement of an additional intracellular xylanase for the cleavage of this substrate. A copy of *xynB1* is present in 17/18 HUS^+^ strains. The presence of a *xynB1* ortholog in *G. caldoxylolyticus* CIC9 suggests its protein product may complement the xylotriose cleaving action of the missing *xynA2* enzyme. In *Geobacillus* sp. JF8, both *xynB1* and *xynA2* orthologs are missing, implying that this strain is incapable of utilizing the xylotriose substrate. Complete copies of *xynB3* are found in 14/18 HUS^+^*Geobacillus* strains. In MAS1, Y412MC52, Y412MC61and C56T3 the *xynB3* reading frame is disrupted by a transposon insertion, but given the similar function of the two β-xylosidases XynB2 and XynB3 in the hydrolysis of xylobiose, the complete *xynB2* copy in these strains may complement the disrupted XynB3 product.

Three intracellular enzymes in the AUS locus of *G. stearothermophilus* T-6 play a role in the concerted release of component arabinose monosaccharides from the arabinan-derived oligosaccharides [21]. The GH51 arabinofuranosidase Abf2 of *B. subtilis* 168 (P94552) has been shown to remove *O*-2 and *O*-3-linked arabinofuranosyl side chains of branched arabinan polymers [60]. The translated protein products of the *abfB* gene found in all four AUS^+^*Geobacillus* strains share 74.9% average amino acid identity with Abf2, suggesting a similar function for AbfB proteins. While the majority of arabinose in plant cell wall polymers exists in the furanose (five-member ring) conformation, a small fraction of arabinose in the side-chain terminal ends of arabinan is in the six-membered ring pyranose form [1, 61]. Orthologs of a GH27 family arabinopyranosidase (Abp) which can cleave the terminal arabinopyranose residues [62] are encoded in the AUS loci of all four strains, but the presence of a transposon integrated in the T-6 *abp* gene suggests this capacity is disrupted in this strain. Finally, the resultant debranched linear α-1,5-arabinofuranose chain is cleaved into its component arabinose monomers by the intracellular GH43 arabinanase AbnB [21, 63], orthologs of which are encoded in all four AUS^+^ strains. Orthologs of a GH51 family arabinofuranosidase (AbfA) are encoded in seventeen HUS^+^ strains. The absence of additional encoded enzymes for the utilization of L-arabinan polymers in most strains suggests that this enzyme plays a role in the liberation of *O*-2 and *O*-3-linked arabinofuranosyl residues from arabinoxylan [21]. The presence of both *abfA* and *abfB* orthologs in the AUS^+^ strains thus suggests that they may have a capacity for debranching both arabinan and arabinoxylan polymers. The HUS locus of C56-T2 lacks orthologs of both *abfA* and *abfB*, but instead carries a gene encoding a distinct GH43 family glycoside hydrolase. The encoded protein shares only 13.5% and 10.7% amino acid identity with the AbfA and AbfB proteins of *G. stearothermophilus* T-6, respectively. However, it shares orthology with the Abf43B protein of *Paenibacillus* sp. E18 (AFC38437; Amino acid identity: 62.8%; Bitscore: 824; E-value: 0.0), which was characterized as a GH43 family α-arabinofuranosidase with a role in degradation of arabinoxylans [64].

In nature, hemicellulose polymers are frequently substituted with acetyl side chains. For example, the main hemicellulose of hardwoods, 4-O-methyl-D-glucuronoxylan, contains acetyl side chains at positions two or three of 70% of the xylose backbone subunits [65]. These acetyl side chains contribute towards the recalcitrance of hemicelluloses by restricting access of hydrolytic enzymes to the backbone sugars. As a ‘counteractive’ measure, many microorganisms produce acetylesterase enzymes, which can cleave the acetyl side chains [66, 67]. Two genes encoding predicted acetylesterases have been identified within the *G. stearothermophilus* T-6 hemicellulose utilization locus, *axe1* and *axe2* [20], with orthologous genes present in 16/18 and 17/18 HUS^+^ strains, respectively. The *axe1* gene forms part of xylooligosaccharide utilization cluster (Figure 1 - cluster G) and the encoded Axe1 protein products share 86.7% average amino acid identity among the HUS^+^ strains. Comparison against the CAZY database with the dbCAN Blast tool shows that they belong to Carbohydrate Esterase family CE4 [41, 68], which incorporates acetylxylan esterases and chitin deacetylases, and show greatest orthology with the predicted polysaccharide deacetylase of *Paenibacillus* sp. JDR-2 (ACT03992.1; Average amino acid identity: 53.4%; Bitscore: 230; e-value: 4.5e-58). The incorporation of the *axe1* gene in the xylooligosaccharide utilization cluster (Figure 1 – cluster G) suggests that this esterase plays a role in the deacetylation of xylan or its internalized derivative oligosaccharides. The crystal structure of the intracellular Axe2 protein of *G. stearothermophilus* T-6 has been determined, and this functionally characterized acetylxylan esterase has been shown to belong to a novel CE family [23, 67]. Orthologs of the Axe2 proteins of sixteen sequenced HUS^+^ strains share 91.2% average amino acid identity with the T-6 Axe2 protein, and negligible sequence similarity to the Axe1 protein in this strain. The *axe2* gene forms part of the second intracellular xylooligosaccharide degradation cluster (Figure 1 – cluster L). While the Axe2 protein has been shown to be active on short acetylated xylo-oligmers [23], the natural target xylooligosaccharide is unknown. The native substrate of Axe1 is also unknown, but it can be hypothesized that these two esterases act in concert to completely deacetylate the xylooligomers resulting from xylan degradation.

A further putative intracellular hydrolytic enzyme is encoded in the HUS locus of JF8, while a transposon-disrupted copy is also found in MAS1. The translated JF8 protein product lacks a recognizable Gram-positive signal peptide, suggesting an intracellular localization, and shares 73% amino acid identity with a GH3 family glycosyl hydrolase of *Thermoanaerobacterium thermosaccharolyticum* DSM571 (Tthe_2487). Furthermore, the amino acid sequence contains a β-glucosidase conserved domain (PRK15098; Bitscore: 284; e-value: 3.7e-84). The predicted JF8 and MAS1 β-glucosidase-encoding (*bgl*) gene is located downstream of *gh5* and *cutDEF* genes, suggesting the encoded enzyme is likely involved in the intracellular hydrolysis of the substrate derived through the extracellular hydrolysis by the GH5 enzyme and intracellular transport by the potentially associated *cutABC* transporter. Orthologs of the JF8 *bgl* gene and the *cutDEF* transporter are, however, absent in *Geobacillus* sp. CAMR12739 and *G. thermoglucosidasius* C56YS93, which contain a partial and complete copy of the GH5-encoding gene, respectively. Members of the GH3 family include glucosidases and xylosidases, and as such the target substrate of this gene cluster and its encoded proteins, whether it is a glucose- or xylose-based polymer, remains undetermined.

#### Variability in the metabolic genes in the HUS loci of Geobacillus spp

The monosaccharides derived from intracellular oligosaccharide cleavage are typically catabolised into intermediate substrates that enter the pentose phosphate and Embden Meyerhof Parnas (EMP) glycolysis pathways [19, 20]. The catabolic systems for D-xylose (*xylAB*), uronic acids (*kdgKA*-*uxaC*-*uxuAB*) and L-arabinose (*araRDBA*) monosaccharides have been well-characterized in *Geobacillus stearothermophilus* and its phylogenetic relative *B. subtilis* [19, 52, 69]. Orthologs of the *xylAB* and *kdgKA*-*uxaC*-*uxuAB* loci are present in all eighteen HUS^+^ strains, while *araRDBA* orthologs are present in seventeen of the strains. The latter locus is absent from *G. thermoglucosidasius* C56Y93, which is also missing orthologs of all additional genes encoding enzymes required for the transport and catabolism of arabinosaccharides, suggesting that the hemicellulose degradation system functions on arabinofuranose-free hemicellulose substrates. In Y412MC52 and Y412MC61, a transposase is inserted into the reading frame of *uxaC*, which encodes a uronate isomerase that catalyzes the first step in the conversion of uronic acids to glycerol-3-phosphate and pyruvate, which can enter the EMP pathway [19]. This suggests that these two strains are unable to catabolise this substrate.

An additional gene cluster, *araJKLMN*, has been identified in the *G. stearothermophilus* T-6 HUS locus. Complete *araJKLMN* loci are present in eleven of the eighteen HUS^+^ strains, while partial or transposon-disrupted loci are present in 6/18 HUS^+^ strains. Both *araK* and *araN* orthologs are absent from strains NG-80, G11MC16 and DSM465, while an *araN* ortholog is missing from the C56-T2 HUS locus. In C56-T3 and CAMR5420, the *araN* reading frame is disrupted by a transposon. A second copy of the *araN* gene, *araN*^*O*^ is located downstream of the *cutABC* locus of GHH01, with its translated protein product sharing 91.3% amino acid identity with the AraN protein in this strain. Similarly, a second copy (*araN*^*O*^) is located upstream of *araPST* in the *G. caldoxylolyticus* CIC9 HUS locus, but its translated product shares lower sequence similarity (57.7% amino acid identity) with the CIC9 AraN protein. The genes in this cluster have been shown to encode orthologs of a NADP sugar dehydrogenase (*araJ*), aldose-1-epimerase (*araK*), sugar phosphatase (*araL*), NADP-dependent glycerol-1-dehydrogenase (*araM*) and hypothetical protein (*araN*) and have been postulated to constitute an alternative pathway for the catabolism of pentose sugars [21].

### Additional genes in the *Geobacillus echD*-*npd*islands may play a role in hemicellulose utilization and alcohol fermentation

Additional non-conserved genes can be observed at the 5′ end of the *echD*-*npd* islands of several strains, flanking the hemicellulose utilization locus (Figure 2). Their translated protein products were annotated by BlastP comparison against the NCBI non-redundant protein database to identify orthologs for which functions have been predicted or experimentally determined (Additional file 3: Table S3). A gene cluster (*dppABCDFE*) at the 5′ end of the *echD*-*npd* island of five HUS^+^*Geobacillus* strains, namely Y412MC52, Y412MC61, MAS1, GHH01 and C56YS93, encodes a predicted oligopeptide transporter. Orthologs could furthermore be identified in the three HUS^-^ strains, *Geobacillus* sp. Y4.1MC1, *G. thermoglucosidasius* TNO-09.20 and M10EXG. The *echD*-*npd* island 5′ end regions of *G. caldoxylolyticus* CIC9, *Geobacillus* sp. G11MC16, and *G. thermodenitrificans* NG80-2 and DSM465^T^ harbor large inserts, 26.6 to 28 kb in size. The genes in these inserts encode a system for the catabolism of the polyol myo-inositol (*iolG*/*IDEBCA*), as well as two predicted ABC sugar transporters. Strains G11MC16, NG80-2 and DSM465^T^ also encode a 1,044 amino acid protein which, by comparison against the CAZY database, was identified as a putative GH38 family glycoside hydrolase. The GH38 protein products show extensive orthology to the functionally characterized α-mannosidase (ManA) of *Bacillus* sp. GL1 (BAB40444.2; Average amino acid identity: 55.6%; Bitscore: 1299; e-value: 0.0; [70]. Mannose monosaccharides frequently form part of the backbone of hemicellulose polymers, such as the galactomannans found in the seeds of members of the *Fabaceae* family, and galactoglucomannans in conifer wood [1]. As such, the presence of a *manA* ortholog and a possible mannose-specific ABC transport system adjacent to the hemicellulose utilization locus, suggests that they form part of the hemicellulolytic arsenal in these strains. However, an ortholog showing 87.9% average amino acid identity to the ManA protein of the three HUS^+^ strains is also incorporated into the *echD*-*npd* island of the HUS^-^ strain *G. kaustophilus* HTA426. The ManA protein may therefore rather form part of the general sugar utilization structure in *Geobacillus* spp. We suggest that the *echD*-*npd* island represents an insertion point for the centralization, and potentially co-regulation, of multiple sugar metabolic pathways in *Geobacillus* spp.

An additional cluster of orthologous genes is also integrated into the HUS loci of *Geobacillus* sp. JF8, MAS1 and CAMR12739 in the non-conserved region harboring the GH5 family glycoside hydrolase-encoding gene (Figure 2; Additional file 1: Table S1). One of these genes codes for an ortholog of the aldehyde dehydrogenase AldH-T of *G. stearothermophilus* SIC1 (BAA02975; Average amino acid identity: 92.2%; Bitscore: 935; e-value: 0.0). This enzyme has been shown to play a role in the conversion of acetyl CoA to aldehydes which are further converted into alcohols by alcohol dehydrogenases [11, 71]. Furthermore, another gene in this cluster encodes an orthologs of the aldo/keto-reductase YtbE of *Bacillus* sp. ECU0013 (ADP24600; Average amino acid identity: 68%; Bitscore: 400; e-value: 4e-114), which has been shown to catalyze the NADH-dependent reduction of aliphatic and aromatic aldehydes into their corresponding alcohols [72, 73]. The co-localization of genes with a putative role in alcohol fermentation and hemicellulose utilization further suggests a role for the *echD*-*npd* island in the centralization, and potentially maintenance and regulation, of the metabolic and fermentative pathways of *Geobacillus* spp.

## Conclusions

Our comparative genomic analyses have revealed the presence of a centralized hemicellulose utilization locus in most sequenced *Geobacillus* strains. However, this locus, which is localized on a common genomic island, displays extensive genetic variability. Genes or pathways central to the utilization of the hemicellulose backbone and substituents are absent from the HUS loci of many strains, while other genes, or operons, are frequently disrupted by transposon insertion. From this we may deduce that different *Geobacillus* strains have a highly variable capacity to degrade hemicellulose polymers. It is possible that, in their natural environments, *Geobacillus* strains form part of consortia, whereby strains deficient in genes or pathways required for hemicellulose degradation and utilization may be complemented by other members of the consortia. However, given the limited number of extracellular hydrolytic enzymes produced by *Geobacillus*, which are generally conserved across their HUS loci, and the specific trans-membrane transport systems they utilize for the internalization of relatively large oligosaccharides, we argue that such complementation is unlikely. Comparisons of the HUS loci of the sequenced strains to that of the well-characterized *G. stearothermophilus* T-6 revealed the presence of a number of additional genes, some of which have orthologs in related genera and code for both extra- and intracellular hydrolytic enzymes and transmembrane transporters. These findings further support the concept of variable hemicellulolytic capacities among *Geobacillus* strains, and imply that they have evolved to degrade distinct hemicellulose substrates, such as are found in different plant species and tissues. For example, the lack of genes encoding an α-glucuronidase (*aguA*), its associated transport system (*aguEFG*) and uronate metabolic genes in *Geobacillus* sp. JF8 implies that this strain utilizes a xylan which is not substituted with glucuronic acid, while a xylan polymer devoid of arabinofuranosyl substituents is the likely target of *G. thermoglucosidasius* C56YS93. This novel substrate-guided ‘host-parasite’ relationship has not been demonstrated experimentally, but is potentially testable. A recent review highlighted the extensive geographical and environmental diversity of members of the genus *Geobacillus* and their important ecological roles in composting of plant biomass under thermophilic conditions [74]. Their ability to degrade distinct hemicellulose polymers may thus reflect their environmental exposure to particular hemicelluloses and potentially, their role in carbon cycling in places where hot conditions persist.

The extensive genetic variability among the *Geobacillus* hemicellulose utilization systems highlighted in this study can potentially serve as a backbone for the development of recombinant *Geobacillus* strain(s) with improved hemicellulolytic capacities or with the capacity to degrade structurally diverse hemicellulose substrates. Such engineered strains, with the added advantage of the thermostability of many of the *Geobacillus* extra- and intracellular hydrolytic enzymes, could then be exploited in various biotechnological applications, such as the production of bioethanol and other value-added products.

## Methods

### Elucidation and characterization of the *Geobacillus*hemicellulose utilization loci

The *G. stearothermophilus* T-6 hemicellulose utilization locus (NCBI Acc. # DQ868502) was subdivided into thirteen gene clusters on the basis of their predicted function. The nucleotide sequences of these clusters, as well as the translated protein products for the genes were compared by localized BlastN and tBlastN using the Bioedit v. 7.1.11 software package [75] to identify orthologs in the partial or complete genome sequences of twenty-four *Geobacillus* strains (Table 1). The Genbank sequences of thirteen complete *Geobacillus* genomes were submitted to the Islandviewer server, which integrates several tools for the prediction of genomic islands [36]. Localized BlastN analysis was subsequently used to elucidate the complete *echD*-*npd* islands in the eleven *Geobacillus* strains for which only partial genome sequences are available and *G. stearothermophilus* T-6. Mean G + C contents for the genome sequences, the *echD*-*npd* islands and for individual genes were determined using Bioedit [75]. Open reading frames were predicted using the Softberry FgenesB server [38] and the resultant CDS sets were standardized by local BlastN and BlastP comparison.

### *In silico*analyses of the HUS locus and *echD*-*npd*island protein coding sequence sets

The translated protein products encoded in the *echD*-*npd* islands and HUS loci were compared to the NCBI non-redundant protein database to identify orthologs which have been characterized functionally and/or *in silico* (Additional file 1: Table S1). Comparisons between the proteins sets of the different *Geobacillus* strains were performed using Localized BlastP analysis in Bioedit [75]. Conserved protein domains were identified by comparison against the NCBI conserved domain database using CD-Search [76, 77]. Signal peptides were predicted using the SignalP 4.1 server [40] and the subcellular localizations of the proteins were determined with the PsortB 3.0 server [39]. Proteins showing orthology to transport-related proteins were compared to the Transport Classification Database (TCDB) [78]. Glycoside hydrolases and carbohydrate esterases encoded in the HUS loci were characterized and classified into their respective families by comparison against the CAZY database using the dbCAN Blast tool [41, 42, 68].

### Phylogenetic analyses

The nucleotide sequences of the *recN* gene, which codes for DNA repair protein RecN, of the twenty-four sequenced *Geobacillus* strains as well as twelve *Geobacillus* spp. type strains were used to demonstrate the phylogenetic relationships among the HUS^+^ and HUS^-^*Geobacillus* strains. Similarly, the RecN amino acid sequences of the HUS^+^ strains and twelve strains belonging to related genera, as well as the concatenated XynDCEFG-XylAB amino acid sequenced were used to construct phylogenies. Sequence alignment was performed using Clustal Omega v. 1.2.0 [79], with default parameters. Phylogenetic trees were constructed with the Molecular Evolutionary Genetics Analysis (MEGA) v.5.0.3 software package [80], using the neighbour-joining method with the following parameters: Poisson correction, complete gap deletion and bootstrapping (n = 1,000).

### Availability of supporting data

The complete genome sequences of all the *Geobacillus* strains incorporated in this study are available on the NCBI database under the NCBI accession numbers listed in Table 1. The phylogenies included in the manuscript (Figures 3 and 4) have been deposited and available in TreeBASE [81]. The amino acid sequence sets for each hemicellulose utilization locus and the Genbank files for each of the representative hemicellulose loci are available in the LabArchives repository [82].

## Electronic supplementary material

Additional file 1: Table S1: Mean G + C contents for the conserved and non-conserved genes in the *echD*-*npd* islands of *Geobacillus* sp. A8 and *G. caldoxylolyticus* CIC9. The mean G + C contents for forty-one genes conserved in both strains and seven and twenty-eight non-conserved genes in strains A8 and CIC9, respectively, were calculated. (DOCX 13 KB)

Additional file 2: Table S2: Annotations of the genes/proteins encoded in the HUS loci of the *Geobacillus* strains. The gene names, as well as the size and predicted functions of the encoded proteins are shown. The presence of an ortholog in a given *Geobacillus* strain is indicated by a black block, while absence of orthologs is denoted by a '-'. The *Geobacillus* strain numbers are those indicated in Table 2. Orthology to proteins in non-*Geobacillus* organisms was determined. (XLSX 130 KB)

Additional file 3: Table S3: Annotations of the additional genes/proteins encoded at the 5′ end of the *echD*-*npd* islands of *Geobacillus* strains. The gene names, as well as the size and predicted functions of the encoded proteins are shown. The presence of an ortholog in a given *Geobacillus* strain is indicated by a black block, while absence of orthologs is denoted by a '-'. The *Geobacillus* strain numbers are those indicated in Table 2. Orthology to proteins in non-*Geobacillus* organisms was determined. (XLSX 31 KB)
