# Supplementary material for: Comparative analysis of the Geobacillus hemicellulose utilization locus reveals a highly variable target for improved hemicellulolysis
Source: BMC Genomics. 2014 Oct 1;15(1):836. doi: 10.1186/1471-2164-15-836 (PMC4194401; doi:10.1186/1471-2164-15-836)
Supplement: Supplementary file 1 — Additional file 1: Table S1: Mean G + C contents for the conserved and non-conserved genes in the echD-npd islands of Geobacillus sp. A8 and G. caldoxylolyticus CIC9. The mean G + C contents for forty-one genes conserved in both strains and seven and twenty-eight non-conserved genes in strains A8 and CIC9, respectively, were calculated. (DOCX 13 KB) [file 12864_2014_6519_MOESM1_ESM.docx]

**Supplementary Table S1 Mean G+C contents for the conserved and non-conserved genes in the *echD-npd* islands of *Geobacillus* sp. A8 and *G. caldoxylolyticus* CIC9.**

| ***Geobacillus* strain** | **A8** | **CIC9** |
| --- | --- | --- |
| Mean Genomic G+C content | 52.41% | 44.17% |
| Mean *echD-npd* island G+C content | 46.75% | 38.83% |
| # conserved genes | 41 | 41 |
| # non-conserved genes | 7 | 28 |
| G+C content conserved | 47.23% | 39.48% |
| G+C content non-conserved | 49.53% | 39.05% |
